# Detection of novel reassortant H9N2 avian influenza viruses in wild birds in Jiangxi Province, China

**DOI:** 10.1002/vms3.391

**Published:** 2021-03-03

**Authors:** Tao Zhang, Ruiyun Li, Pinghua Zhong, Jianyu Chang, Bing Xu

**Affiliations:** ^1^ Ministry of Education Key Laboratory for Earth System Modeling Department of Earth System Science Tsinghua University Beijing China; ^2^ Centre for Healthy Cities Institute for China Sustainable Urbanization Tsinghua University Beijing China; ^3^ MRC Centre for Global Infectious Disease Analysis Department of Infectious Disease Epidemiology School of Public Health Faculty of Medicine Imperial College London London United Kingdom; ^4^ Jiangxi Environmental Engineering Vocational College Jiangxi China; ^5^ College of Veterinary Medicine China Agricultural University Beijing China

## Abstract

The five avian influenza A/H9N2 viruses isolated from wild birds in Jiangxi, China in 2015 are novel reassortants which most likely evolved from multiple lineages. They shared a high similarity with isolates from poultry, suggesting a frequent contact and continuous viral circulation at the bird‐poultry interface. Given the continuous reassortment of H9N2 viruses, it will of substantial importance to implement routine surveillance in wild birds to successfully control avian influenza viruses and better the early warning system of the emerging reassortants with pandemic potential.

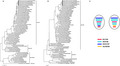

Avian influenza A virus (H9N2) has become widespread and established a stable lineage in domestic poultry in China since its first identification in Guangdong Province in 1994 (Chen et al., 1994). Sporadic spillover to humans has also been documented (Butt et al., 2005; Pan et al., 2018; Peiris et al., 1999). Phylogenetic analysis revealed that H9N2 viruses have undergone frequent genetic reassortment, leading to a high phylogenetic diversity and distinct lineages (Dong et al., 2009; Wang et al., 2016). Additionally, H9N2 have facilitated the emergence of novel reassortant H7N9, H5N6 and H10N8 viruses by providing internal genes, posing a substantial threat to public health (Bi et al., 2016; Chen et al., 2014; Liu et al., 2015; Pu et al., 2014; Shen et al., 2016; Yu et al., 2013). These findings are suggestive of the ability and potential of H9N2 to generate new reassortant viruses with pandemic potential in humans.

Frequent exposure and viral transmission between wild birds and domestic poultry may support the continuous circulation of viruses in the avian reservoir. Wild birds and waterfowl are the natural hosts of AIVs (Alexander, 2000, 2007) distributing viruses to remote areas via long‐distance migration. Domestic poultry may become infected through direct contact with infected waterfowl or other infected poultry, or through frequent exposure to contaminated surfaces (CDC, 2019).

To better understand the evolution of H9N2 viruses, 45 tracheal and cloacal swab samples were collected from wild birds in Suichuan county in Jiangxi Province, China, during the routine bird banding survey in 2015. These specimens were preserved in a sample solution in the fridge (4℃) and subsequently shipped to the laboratory and stored frozen at −80℃. Virus isolation was conducted in 9‐ to 11‐day‐old specific pathogen‐free (SPF) embryonated chicken eggs. Five out of 45 samples had haemagglutination activity. The viral RNAs of these positive samples were extracted from allantoic fluid with haemagglutination activity using RNeasy Mini Kit (Qiagen,). The superscript III reverse transcription‐PCR (RT‐PCR) kit (Invitrogen) was used for the reverse transcription synthesis viral cDNA. All segments were amplified using a Phusion high‐Fidelity PCR system (New England Biolabs) adhering to the manufactory guide. Full‐genome sequencing was performed with Applied Biosystems Automated 3730xl DNA Analyzer. subtype. All animal works are in according with the Animal Care and Use Committee guidelines of China Agricultural University (SKLAB‐B‐2010‐003).

To investigate the phylogenetic characterization of H9N2 viruses, phylogenetic trees of eight genes were constructed using the maximum likelihood method based on the Kimura 2‐parameter model (Kimura, 1980) in MEGA 6.0 (Tamura et al., 2013). Reference sequences were downloaded from the GenBank database of the National Centre for Biotechnology Information. The majority (>90%) of these sequences were isolated from poultry, whereas the rest were from wild birds and human beings. To classify H5N1 and H9N2 lineages, sequences before 2000 from outside China were also included. The representative virus of each lineage was provided in Table S9.

The isolation rate of H9N2 viruses was 11% (5 H9N2 positive samples out of 45 samples). H9N2 positive samples were collected from Chinese Pond Heron (*Ardeola bacchus*), Yellow Bittern (*Ixobrychus sinensis*) and Striated heron (*Butorides striatus*). They are designated as A/Chinese Pond Heron/Jiangxi/K64/2015(H9N2), A/Yellow Bittern/Jiangxi/K65/2015(H9N2), A/Striated Heron/Jiangxi/K66/2015(H9N2), A/Chinese Pond Heron/Jiangxi/K76/2015(H9N2) and A/Yellow Bittern/Jiangxi/K77/2015(H9N2). Phylogenetic analyses showed that all five isolates belonged to the H9N2 subtype. Notably, there is a distinct phylogenetic structure in each gene segment, with lineages derived from H9N2 and H5N1 (Figure 1a and b; Figure S1). Amongst these lineages, SH/F/98(H9N2) was the most prevalent lineage whose NA, PB1, PA and NP genes are closely related to the five isolated viruses in Jiangxi. Of note, M gene segment showed a close relationship with two lineages, that is HK/G1/97(H9N2) and Gs/GD/96(H5N1) (Figure 1c). This phylogenetic structure suggests a more complex reassortment process of the five Jiangxi H9N2 isolates compared with previous H9N2 variants (Gu et al., 2019). The ability of reassortment with lineages from high pathogentic subtypes including H5 and H7 (Bi et al., 2016; Pu et al., 2014) highlighted its potential threats to public health.

**FIGURE 1:**
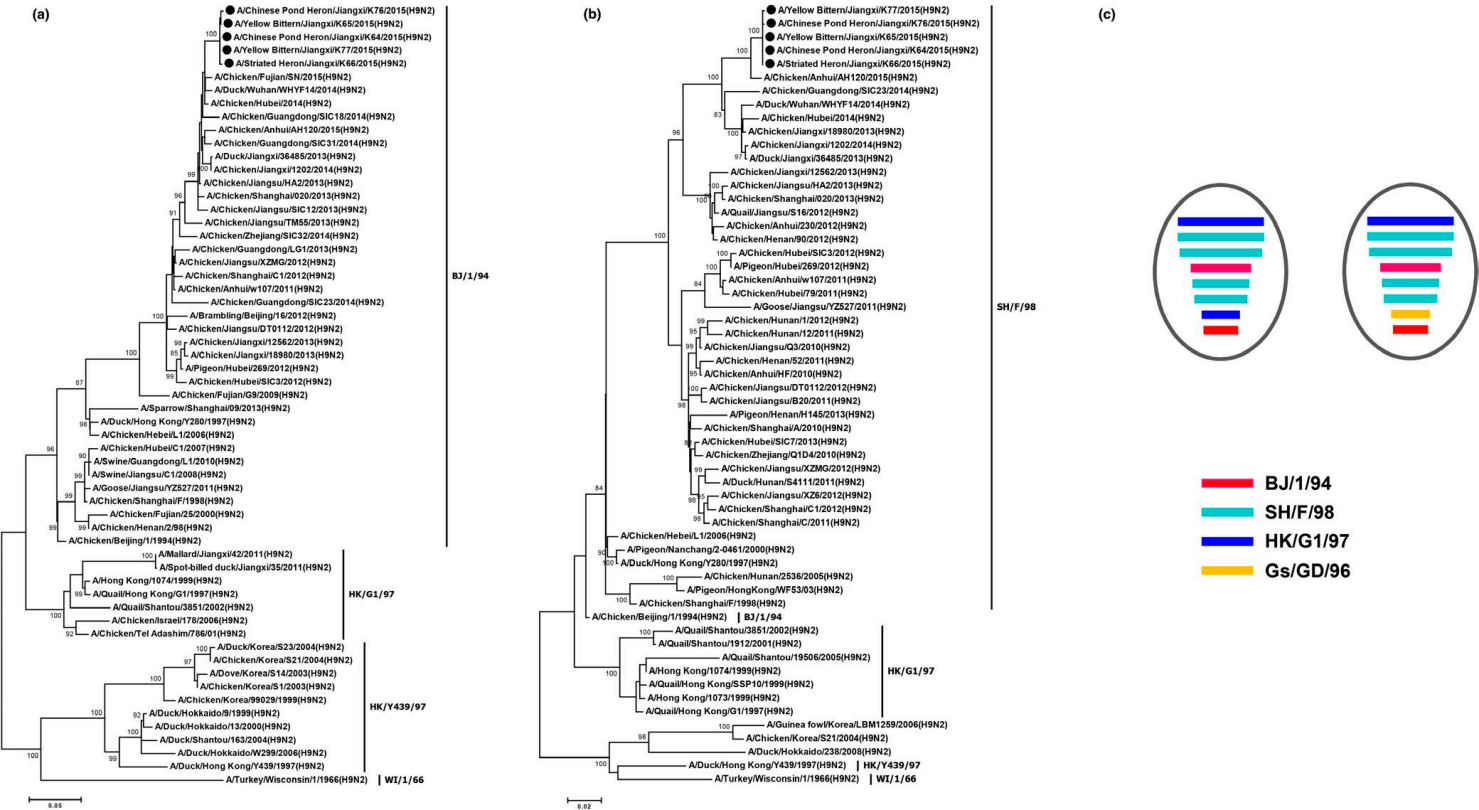
Phylogenetic analysis of five viruses isolated in Jiangxi, China in 2015. Molecular phylogenetic analyses for the (a) HA, (b) NA were conducted using the Maximum Likelihood method based on the Kimura 2‐parameter model. Viruses sequenced in this study were marked by circles. Internal branching probabilities were determined by bootstrap analysis with 1,000 bootstrap replicates. Viruses formed distinct groups, that is A/chicken/Beijing/1/94(H9N2) (BJ/1/94), A/chicken/Shanghai/F/98(H9N2) (SH/F/98), A/quail/Hongkong/G1/97(H9N2) (HK/G1/97) and A/goose/Guangdong/1/96(H5N1) (Gs/GD/96). (c) A hypothetical reassortment pattern of the novel H9N2 virus isolates. The eight gene segments (horizontal bars), that is PB2, PB1, PA, HA, NP, NA, M and NS, were ordered from top to bottom in each virion. Different colours represented different virus lineages

To address the genetic similarity between viruses isolated from wild birds and domestic poultry, average genetic distance, defined as the number of base substitutions per site from averaging over all sequence pairs, was used to represent the evolutionary divergence between two species. Analysis were calculated using the Kimura 2‐parameter substitution model with a gamma‐distributed variation rate among sites (shape parameter = 4).

The average genetic distance between domestic poultry and wild birds vary from 0.001 to 0.105 substitutions per site depending on the segment (Table 1). This minor difference is suggestive of a minimal evolutionary divergence between species, which may due partly to the frequent mutual transmissions between domestic and wild birds. Note that the size of samples within wild birds is limited. Therefore, genetic distance within and among species should be interpreted with caution. First, the small genetic distance within birds in NA gene indicates a typically high genetic similarity among viruses in birds. This similarity, however, should not be interpreted as a general characteristic of viral evolution with birds. Furthermore, genetic distance within birds is not comparable with that within poultry due primarily to the different sample size and spatial‐temporal coverage of samples between two species.

**TABLE 1:** Estimates of evolutionary divergence over sequence pairs within and between groups

Gene segment	Birds		Poultry		Birds‐poultry	
	Mean	*SE*	Mean	*SE*	Mean	*SE*
HA	0.087	0.005	0.079	0.004	0.088	0.006
NA	0.001	0.001	0.073	0.004	0.079	0.005
PB2	0.086	0.006	0.112	0.007	0.105	0.007
PB1	0.083	0.004	0.054	0.002	0.076	0.004
PA	0.089	0.006	0.097	0.006	0.095	0.006
NP	0.071	0.005	0.071	0.004	0.076	0.004
M	0.069	0.005	0.035	0.003	0.053	0.004
NS	0.037	0.005	0.065	0.006	0.064	0.006

Note

H9N2 sequences isolated in China in 2000–2015 are assigned to either domestic poultry or wild birds group. Evolutionary divergence is evaluated using the average genetic distance, or the number of base substitutions per site from averaging over all sequence pairs within or between groups. Standard error (*SE*) estimates are obtained by a bootstrap procedure with 1,000 replicates. Analysis is calculated using the Kimura 2‐parameter substitution model with a gamma‐distributed variation rate among sites (shape parameter = 4). All codon positions containing gaps or missing data are eliminated. The number of positions of each segment in the final dataset is HA: 1549, NA: 1,343, PB2: 1,213, PB1: 2,156, PA: 1,630, NP: 1,428, M: 906 and NS: 628.

The phylogenetic diversity of H9N2 isolates reflected the role that migratory birds played in viral evolution and transmission. It is recognized that China provides a suitable condition for gene exchange among different host species and regions, giving rise to novel H3N8 and H5N6 Clade 2.3.2.1c reassortants (Li et al., 2019; Zhang et al., 2019). The diverse natural ecological landscape in Suichuan county contributes to the congregation and hence gene exchange among different bird species along the East Asia‐Australia migratory flyway (Xiao et al., 2006). Moreover, the traditional free‐range backyard approach of poultry raising has created an ideal situation for the exposure to the infected birds or environment and viral transmission to poultry (Figure 2). Likewise, previous studies of bird migration across Qinghai and Poyang Lake uncovered the linkage between wild migratory birds and the spread of H5N1 viruses (Cui et al., 2011; Prosser et al., 2011; Takekawa et al., 2010).

**FIGURE 2:**
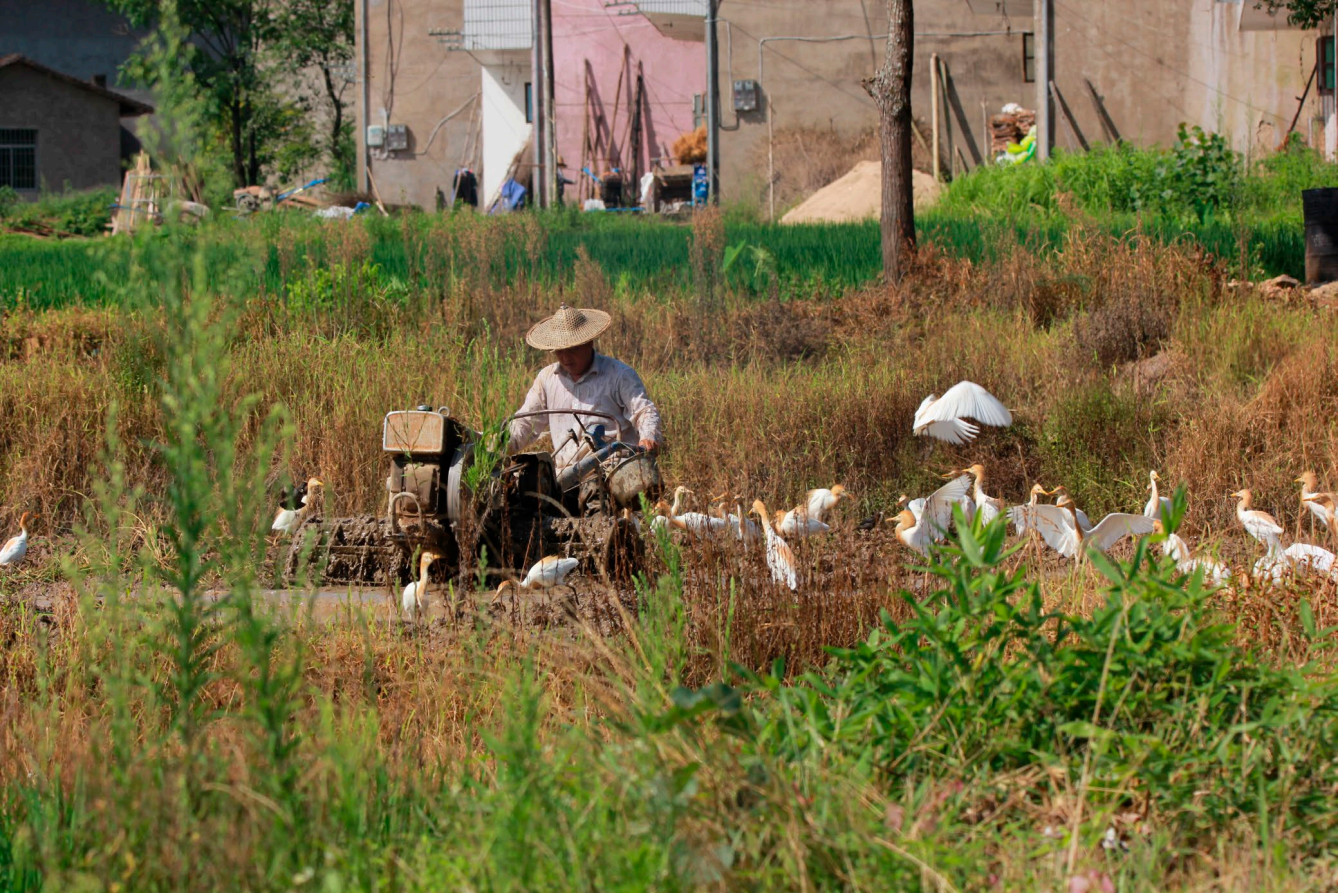
Stopover site of migratory birds in Suichuan county. Migratory birds concentrated in the rice fields surrounding the house where domestic poultry were raised by a free‐range approach, providing an environment for bird‐poultry interaction and inter‐species viral transmission

Overall, the isolation of novel H9N2 reassortants in wild birds highlighted that extensive surveillance of H9N2 should be implemented to improve the early warning of the emergence of novel reassortants with pandemic potential.

## CONFLICT OF INTEREST

We do not have any financial or other relationships that may pose conflicts of interest.

## AUTHOR CONTRIBUTION

**Tao Zhang:** Data curation; Investigation; Methodology; Writing‐original draft; Writing‐review & editing. **Ruiyun Li:** Conceptualization; Methodology; Writing‐original draft; Writing‐review & editing. **Pinghua Zhong:** Data curation; Investigation. **Jianyu Chang:** Data curation; Investigation; Supervision. **Bing Xu:** Conceptualization; Supervision; Writing‐original draft; Writing‐review & editing.

## PEER REVIEW

The peer review history for this article is available at https://publons.com/publon/10.1002/vms3.391.

## DATA AVAILABILITY STATEMENT

Sequence data have been deposited into the GISAID platform (http://platform.gisaid.org/) with the accession numbers of EPI1485062‐EPI1485079 and EPI1485083‐EPI1485104.

## Supporting information

FigS1‐TableS1
