# Supplementary material for: Detection of novel reassortant H9N2 avian influenza viruses in wild birds in Jiangxi Province, China
Source: Vet Med Sci. 2021 Mar 3;7(3):1042–6. doi: 10.1002/vms3.391 (PMC8136960; doi:10.1002/vms3.391)
Supplement: Supplementary file 1 — FigS1‐TableS1 [file VMS3-7-1042-s001.docx]

**Supplementary Information**

**Supplementary Figures**

Figure S1. Phylogenetic analysis of the internal genes of five viruses isolated in Jiangxi, China in 2015. Molecular phylogenetic analyses for the (A) PB2, (B) PB1, (C) PA, (D) NP, (E) M and (F) NS genes were conducted using the Maximum Likelihood method based on the Kimura 2-parameter model. Viruses isolated in this study were highlighted using circles. Lineage classification were in accordance with HA and NA genes provided in Figure 1.


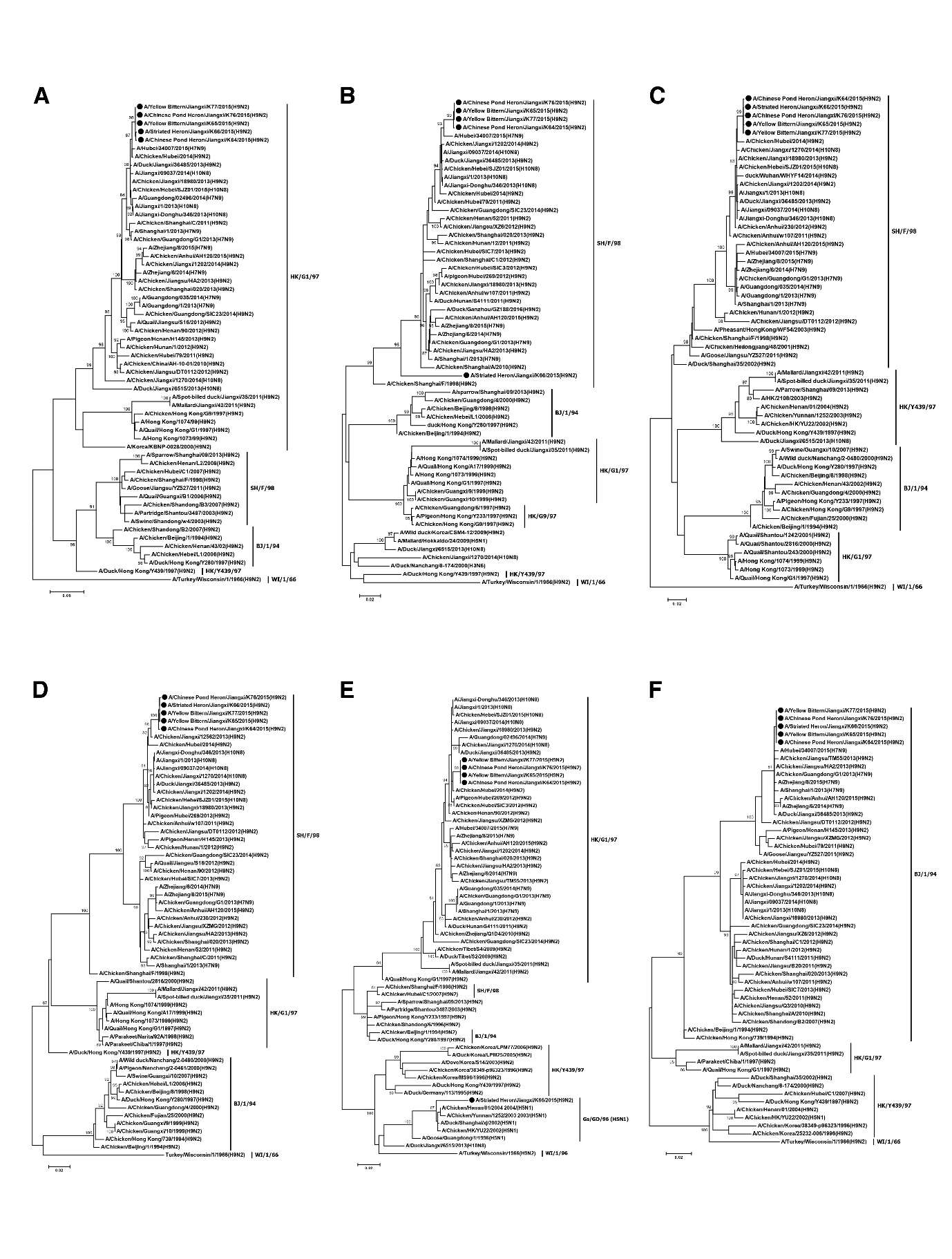


**Supplementary Tables**

Table S1. Representative virus of H9N2 and H5N1 lineages.

| Lineage | Representative virus |
| --- | --- |
| BJ/1/94 | A/chicken/Beijing/1/94(H9N2) |
| HK/Y439/97 | A/duck/Hongkong/Y439/97(H9N2) |
| SH/F/98 | A/chicken/Shanghai/F/98(H9N2) |
| HK/G1/97 | A/quail/Hongkong/G1/97(H9N2) |
| WI/1/66 | A/turkey/Wisconsin/1/1966(H9N2) |
| Gs/GD/96 | A/Goose/Guangdong/1/1996(H5N1) |
